# Sitagliptin/Metformin Versus Insulin Glargine Combined With Metformin in Obese Subjects With Newly Diagnosed Type 2 Diabetes

**DOI:** 10.1097/MD.0000000000002961

**Published:** 2016-03-18

**Authors:** Ming Ji, Libin Xia, Jingzhu Cao, Dajin Zou

**Affiliations:** From the Department of Endocrinology,Shanghai Changhai Hospital,No.168 Changhai Road,Shanghai 200433,China (MJ, JC, DZ) and Department of Endocrinology, Yijishan Hospital of Wannan Medical College,Wuhu,Anhui,241000,China (LX, DZ), China.

## Abstract

To compare the therapeutic effects of different regimens in Chinese obese type 2 diabetic mellitus (T2DM) patients.

From October 2013 to July 2014, a total of 166 T2DM outpatients who attended the Shanghai Changhai Hospital and the Yijishan Hospital of Wannan Medical College were randomly assigned into an experimental sitagliptin/metformin combined with low caloric diet group (n = 115) and an insulin glargine combined with metformin control group (n = 51). Inclusion criteria were body mass index (BMI) ≥ 25 kg/m^2^ and diagnosed with T2DM with glycosylated hemoglobin (glycated hemoglobin A1C [HbA1c]) >9%. Main outcome parameters were fasting plasma glucose, postprandial plasma glucose, BMI, HbA1c, fasting C-peptide, 2-h postprandial C-peptide, triglyceride (TG), total cholesterol (TC), high-density cholesterol (HDL-C), and low-density cholesterol (LDL-C), which were determined by the 75 g steamed-bun meal tolerance test before and 4, 8, 12, and 24 weeks after the treatment started. Treatment costs and life quality were also assessed.

BMI, HbA1C, TG, TC, and LDL were significantly more reduced (*P* < 0.000) and HbA1c significantly better improved in the experimental group than in the control group (<6.5% in 24 [20.87%] vs 2 [3.92%], *P* < 0.001; <7% in 65 [56.52%] vs 12 [23.53%], *P* < 0.001). Quality of life scores in the experimental group increased more than in the control group (*P* < 0.001). The costs for the experimental group medication were less than for other regimens.

For obese T2DM patients diagnosed with a glycosylated hemoglobin level >9%, oral sitagliptin/metformin combined with a low caloric diet effectively and economically maintained glycemic control and significantly improved life quality.

## INTRODUCTION

Obesity or being overweight has always been an independent risk factor for multiple disorders, especially diabetes, as a consequence of a variety of active factors secreted by adipose tissue. These factors cause insulin resistance (IR) as well as various adverse effects on glucose metabolism.^1,2^ IR has been linked to lipid metabolism disorders such as hypertriglyceridemia and high blood-free fatty acid levels.^3^ In addition, obesity can readily predispose patients to hypertension, dyslipidemia, coagulation abnormalities and fibrinolysis disturbance, inflammatory reactions, and so on, eventually causing damage to vascular endothelial cells, leading to the development of atherosclerosis and an increased risk of serious cardiovascular disease.^4^ In recent years, increasing young and obese patients have been diagnosed with type 2 diabetic mellitus (T2DM) and early insulin injection has been recommended to achieve glycemic control,^5^ because early intensive insulin therapy can help to restore and maintain the functions of β cells in newly diagnosed T2DM patients.^6^ But for obese or overweight newly diagnosed T2DM patients who have glycated hemoglobin A1C (HbA1c) levels >9.0%, multiple approaches including lifestyle intervention, hypoglycemic drugs such as incretin and intensive insulin treatment, are all considered to be important components of a comprehensive therapeutic plan. It is still debated which therapeutic approach should be used as first choice therapy in these patients. The Chinese T2DM treatment guidelines (2013 edition) recommends metformin or alpha-glycosidase inhibitor/insulin secretagogues as the first-line therapy and alpha-glycosidase inhibitor/insulin secretagogues, thiazolidinedione, and DPP 4 inhibitors as second-line therapy. Insulin is included in the third- and fourth-line therapy choices. Further treatment will be considered only when previous therapies for glycemic control have failed (HbA1c ≥ 7.0%). Therefore, oral hypoglycemic drugs are still among the first therapeutic choices for Chinese patients with T2DM. Results from meta-analysis including the ACCORD, VADT, ADVANCE, and UKPDS studies have shown that improved glycemic control can reduce the risk of major vascular events by 9% but increase the risk of hypoglycemia due to adverse events. Moreover, the increase in the variability of blood glucose levels in T2DM patients also increases the risk of cardiovascular events and death; thus, smooth hypoglycemic intervention is critical. This intervention is worthy of attention among those obese or overweight diabetic patients because obesity is regarded as an important predisposing factor for IR. Excessive fat deposition in skeletal muscle, adipose tissue, and the liver will lead to systemic IR and related inflammatory reactions. A study in Singapore revealed remarkable differences in insulin sensitivity between individuals with similar body mass indices (BMIs), with ethnicity being 1 of the key factors. For example, the IR of Asian people is more severe than people of difference races who have similar BMIs. A study from Japan found that in Asian T2DM patients,^7^ the glycogen output was significantly higher in patients with normal body weight compared with those who were obese, and their risks of cardiovascular (CV) events were remarkably reduced. Thus, the characteristics of Asian people need to be taken into account when planning therapy, considering the higher CV risks found in Asian people compared to white people with a similar BMI.

A recent report from JAMA showed that the use of exogenous insulin too early could promote CV disorders or even death.^8^ The potential mechanism might be related to weight gain caused by the anabolic role of excessive insulin in peripheral tissues. However, an appropriate controlled study focusing on optimizing treatment strategies in these patients has not been carried out, especially in obese Chinese T2DM patients. Thus, we conducted this study to explore the optimal therapeutic strategies for newly diagnosed T2DM patients who were overweight or obese and had a glycosylated hemoglobin level >9%. A comprehensive comparison between oral sitagliptin/metformin and insulin glargine combined with oral metformin was performed by analyzing basic clinical characteristics, serum lipids, quality of life, and pharmacoeconomical measurements.

## PATIENTS AND METHODS

### Study Subjects

A total of 166 newly diagnosed T2DM patients who came to the outpatient clinic of the Endocrinology Department in the Shanghai Changhai Hospital from October 2013 to July 2014 were enrolled in the study. The diagnosis of T2DM was established according to the T2DM diagnosis criteria (WHO 1999). Inclusion criteria were a disease course of <1 year, BMI ≥ 25 kg/m^2^, HbA1c levels > 9%, fasting C-peptide (FCP) > 2.0 ng/mL, and patients older than 18 years of age. Exclusion criteria were acute diabetic complications such as infections, severe impairment of hepatic or renal functions or cardiac insufficiency, as well as the usage of hypoglycemic drugs and metformin contraindications.

Written informed consent was obtained from each of the participants. Exclusion criteria were acute diabetic complications such as infections or a stressed state and severe impairment of hepatic or renal functions or cardiac insufficiency.

### Randomization

We randomly assigned a total of 166 participants (102 males, 64 females, mean age 54.1 ± 12.5 years) into 2 groups in a 2:1 ratio: experimental group: 115 cases, 71 males and 44 females, mean age 54.1 ± 12.5; control group: 51 cases, 31 males and 20 females, mean age 53.5 ± 11.1. All participants completed the follow-up examinations.

### Interventions

All patients were on a diet during the study period and the daily kcal uptake was ≤1400 with no carbohydrates for dinner.

### Experimental Group

A fixed-dose combination (FDC) of 50 mg sitagliptin and 850 mg metformin (Janumet; Merck Sharp & Dohme, Shanghai, China) was administered orally at breakfast, 1 tablet a day for the first week. If no gastric discomfort was reported, the dose was increased to 1 tablet at both breakfast and dinner.

### Control Group

Insulin glargine administration was initiated with 10 U subcutaneous injections before sleep once a day and then adjusted according to the measured blood glucose values. The final dosages ranged from 10 to 18 U and the mean was 14.4 ± 3.2 U. Meanwhile, 850 mg metformin was used orally once a day for the first week. Similarly, if no gastric discomfort was reported, the dose was increased to 1 tablet at both breakfast and dinner. Atorvastatin (20 mg) was administered to all participants with a low-density lipoprotein cholesterol (LDL-C) level > 2.6 mmol/L.

### Measurement of Baseline Characteristics and Clinical Indices

Baseline characteristics data were collected at admission including: age, gender, past medical history, personal history, family history, BMI, waist circumference (WC), disease course, etc.

The comparable study of multiple clinical indexes between the 2 groups was made before and 4, 8, 12, and 24 weeks after treatment, including the 75 g steamed bread test (fasting blood glucose [FBG] and postprandial blood glucose [PBG], mmol/L), BMI, HbA1C, triglyceride (TG), TC, high-density lipoprotein (HDL), LDL, FCP, 2-h postprandial C-peptide (2hCP), serum total bilirubin (STB), alanine aminotransferase (ALT), gamma glutamyl transferase (γ-GT), blood urea (BU), serum creatinine (SCr), and uric acid (UA).

Three to five milliliter of elbow venous blood was collected from all of the patients and serum separation was performed within 2 h. Then we carried out an electrochemiluminescence immunoassay (Roche, Shanghai, China) to measure the concentrations of C-peptide (intra-assay coefficient of variation was 4.55%; inter-assay coefficient of variation was 5.36%).

Measurements of other clinical indices were conducted as follows: glucose oxidase method for measuring serum glucose, high-pressure liquid chromatography for measuring HbA1c and enzymatic measurement of TC.

### Life Quality Scoring

We quantified a patient's life quality based on a 36-item short form health survey (SF-36),^9^ which consisted of 36 individual events and was based on 8 aspects. The centesimal scores were collected for each patient by evaluating multiple dimensions of life quality: general health, physical functions, physical role, body pain, vitality, social function, emotional role, and mental health. Each dimension had up to 100 points, and the higher a patient scored the better their life quality was assessed.

### Statistical Analysis

Statistical analysis of the experimental data was performed with SPSS for Windows (Version 16.0, SPSS Inc., Chicago, IL). Data that was normally distributed are presented as the mean ± standard deviation and categorical variables are expressed as percentages. We conducted independent-sample *t* tests to compare the mean values of 2 samples, while a paired *t* test was used to compare paired data. A chi-squared test was used to compare the rates of 2 samples, and variance analysis was conducted to compare the mean values of multiple samples. *P* < 0.05 was considered to be statistically significant. Data with a skewed distribution are expressed as median and quantiles and significance differences were determined using a Wilcoxon rank test with *P* < 0.05 being considered to be statistically significant. For comparing treatment regimens, group sample sizes of 112 and 56 achieved 80% power to detect noninferiority using a 1-sided, 2-sample *t* test. The margin of noninferiority was −0.010. The true difference between the means is assumed to be 0.400 and the significance level (alpha) of the test is 0.050. The data are drawn from populations with standard deviations of 1.000 and 1.000. The null hypothesis is that the experimental treatment effect is inferior to the standard treatment. The study would have 80% power to reject the null hypothesis with a sample size of 188 participants; 130 in the experimental group and 58 in the control group. This calculation allows for about 10% attrition from both groups; this 10% includes those potentially lost to follow-up or not adhering to the treatment protocol.

## RESULTS

### Baseline Characteristics of the Research Subjects

**FIGURE 1:**
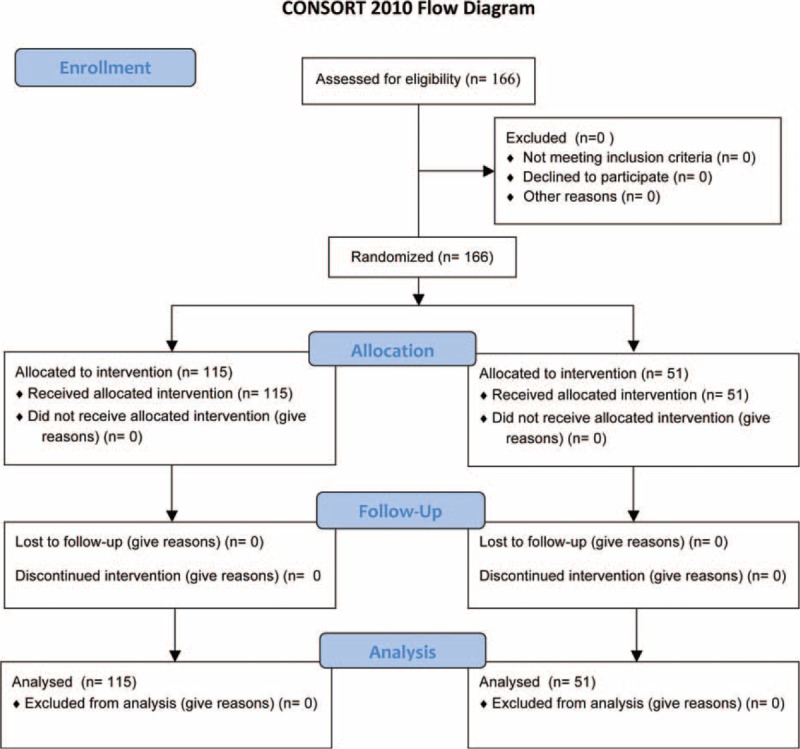
Flow chart of the study.

There were finally 51 cases in the control group (31 males and 20 females, mean age 53.5 ± 11.1) and 115 cases in the experimental group (71 males and 44 females, mean age 54.1 ± 12.5; Figure 1). No significant differences were found in either gender composition or age distribution, HbA1c% and FCP concentrations, whereas BMI and WC were significantly higher in the control group, but fasting plasma glucose concentrations were significantly higher in the experimental group (Table 1).

**TABLE 1:**
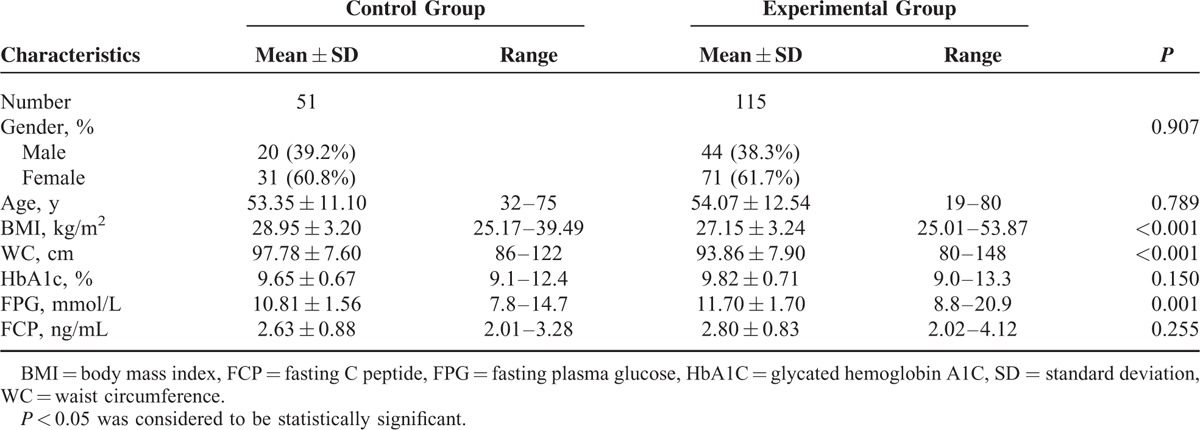
Baseline Characteristics of 166 Patients

### Glycemic Profiles

In both the experimental group and the control group, FBG and 2 h-PBG were significantly reduced at all time points studied (4, 8, 12, and 24 weeks) examined after the corresponding treatment (Table 2). However, there was no significant difference in 2h-PBG between the 2 groups, but a significant difference in FBG was found between the experimental and control groups.

**TABLE 2:**
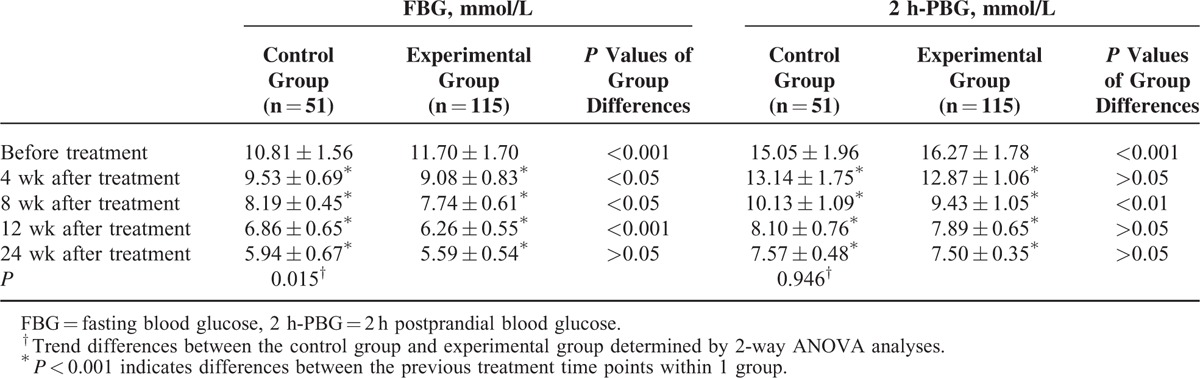
Glycemic Changes After Corresponding Treatment in the Experimental and Control Groups

### Other Serum Indices

Measurements of BMI, HbA1C, TG, and LDL changes (Δ2) after treatment were obviously reduced in the experimental group compared with the control group (*P* < 0.000), but the changes in HDL, FCP, and 2 h-PCP before and after treatment in the experimental group were significantly increased compared with the control group (*P* > 0.05, Table 2). STB, ALT, γ-GT, BU, SCr, and UA concentration changes did not differ significantly between the 2 groups (Table 3).

**TABLE 3:**
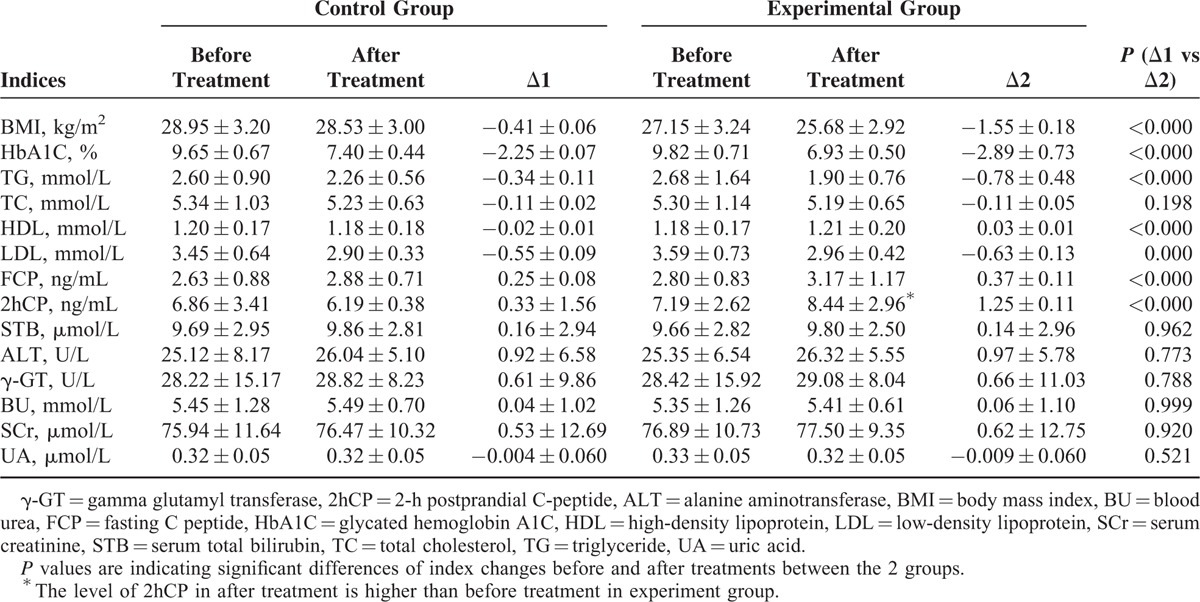
Changes of Other Serum Indices After Corresponding Treatment in the Experimental and Control Groups

### Glycated Hemoglobin (HbA1c) Measurements

Measurements of HbA1c were improved significantly (*P* < 0.000) in the experimental group compared with the control group (Tables 3 and 4). HbA1c was ≤6.5% in 24 (20.87%) of the subjects from the experimental group, while only 2 (3.92%) patients in the control group had their HbA1c reduced to a level ≤6.5% (*P* = 0.017). The HbA1c concentrations in 65 (56.52%) subjects in the experimental group were ≤7.0%, while in the control group the percentage was only 23.53% (12 patients; *P* = 0.012; Table 4).

**TABLE 4:**
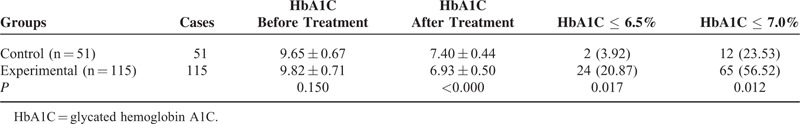
Changes of HbA1c After Corresponding Treatment in the Experimental and Control Groups

### Adverse Reactions

Only 2 cases (1.73%) presented with hypoglycemia out of a total of 115 patients in the experimental group and 4 cases (7.84%) in the control group.

In the experimental group, hypoglycemia occurred in 2 patients (1.73%) with symptoms of cold sweat, heart palpitations, and discomfort. The first hypoglycemia event was at 22:00 pm and the blood glucose concentration was 3.4 mmol/L, whereas the other hypoglycemia occurred at 00:30 am with a blood glucose concentration of 3.6 mmol/L. In the control group, hypoglycemia occurred in 4 patients at 22:00 pm, 1:30 am 10:30 am, and 3:00 am, with symptoms of cold sweat, heart palpitations, hand tremor, and blood glucose concentrations of 3.4, 3.2, 3.8, and 2.9 mmol/L, respectively. The incidences of other adverse reactions were reduced in the experimental group, reaching significance for gas production (*P* < 0.05; Table 5).

**TABLE 5:**
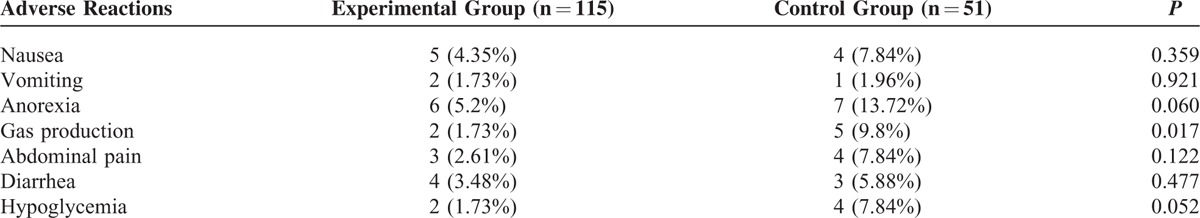
Adverse Reactions After Corresponding Treatment in the Experimental and Control Groups

### Pharmacoeconomical Cost-Effectiveness Analysis

The total cost of drug treatment refers to direct and indirect costs.^10^ Direct costs include the treatment cost together with the cost of treating adverse reactions, and the treatment cost includes charges for examinations, medication, hospitalization, insulin injections, glucose test strips, possible hypoglycemic management, etc. Only outpatients were included in the present study, which thus avoided hospitalization costs. Laboratory examinations and treatments were charged based on the actual costs of drugs: experimental group: Janumet (Merck, 28 tablets/box, 158 yuan RMB/box, 5.65 yuan RMB/tablet) 11.3 yuan RMB/d; control group: Glucophage (Sino American Shanghai Squib Pharmaceutical Ltd., 0.85 × 20 tablets/box, 37 yuan RMB/box, 1.85 yuan RMB/tablet) 3.7 yuan RMB/d; insulin glargine (Beijing Sanofi, 300 U/injection, 237 yuan RMB/injection) 14 U/d and 11.06 yuan RMB/d; Novo Nordisk needles (7 per box, 19.5 yuan RMB/box) 2.79 yuan RMB/needle, Roche glucose test strips, needles (50 pairs/set, 260 yuan RMB/set) 5.2 yuan RMB/pair. All of these add up to the total costs as: 3.7 + 3.7 + 2.79 + 5.2 = 22.75 yuan RMB/d. Indirect costs generally refer to the salary loss of the patient when visiting the doctors, which was negligible in our study because only outpatients were included. Therefore, the therapeutic regimens consisting of Janumet in the experimental group, which showed that the cost was much less than other therapies.

### Quality of Life Score

According to the statistical analysis, multiple dimensions in the quality of life scores (QLSs) showed significant differences between both groups after corresponding treatment, including general health, physical functions, body pain, vitality, social function, mental health, and composite scores (*P* < 0.001). Moreover, the scores of body pain and social function were significantly higher in the experimental group compared with the control group (*P* < 0.05), indicating that the therapeutic regimen in the experimental group was superior to that of the control group. Analysis of variance was performed for repeated measurement data of both groups before and after the treatment. The score after treatment was higher in each group, suggesting that either therapeutic regimen was effective. However, the score in the experimental group increased more than in the control group (*P* < 0.001) and the improvement of quality of life was more prominent in the experimental group compared with the control group (Table 6).

**TABLE 6:**
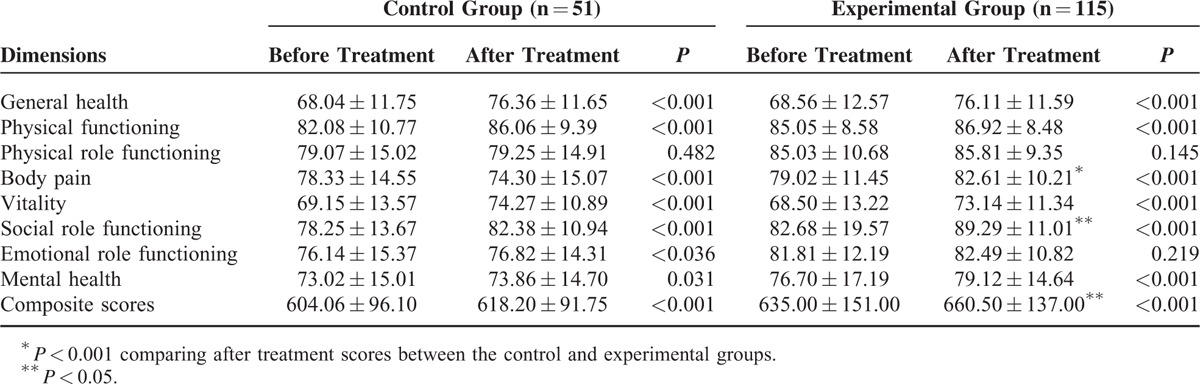
Quality of Life Score Before and After Corresponding Treatment in the Experimental and Control Groups

## DISCUSSION

T2DM is a progressive disease characterized by IR, impaired insulin secretion, increased glycogen dysplasia, and obesity, the incidence of which has been rising consistently worldwide. It is widely recognized to be caused by impaired functions of pancreatic β and α cells.^11^ According to a British prospective study, the functions of pancreatic β cells has been decreasing at a rate of 4% to 5% each year in diabetic patients, even 10 to 15 years before the diagnosis was made.^12^ Mohan et al^13^ conducted an 18-week clinical trial with a single drug for Asian T2DM patients and found that sitagliptin significantly reduced HbA1c by 1% in T2DM patients. In our study, newly diagnosed T2DM patients with HbA1c > 9% and BMI ≥ 25 kg/m^2^ were treated either with oral Janumet (the experimental group) or insulin injection (the control group). HbA1c was ≤6.5% in 20.87% of the subjects in the experimental group but only 3.92% of patients in the control group had their HbA1c reduced to that level. HbA1c measurements of 56.52% subjects in the experimental group were ≤7.0%, while for the control group, the percentage was 23.53. The difference of goal achievements might be attributed to the fact that insulin titration was not optimal due to fear of hypoglycemia particularly in patients on a low carbohydrate diet. In contrast, the DPP-4 inhibitor sitagliptin has not been reported to cause hypoglycemia and its FDC does not need a titration period. Metformin activates the AMPK pathway directly and inhibits gluconeogenesis and lipid synthesis. Through the insulin signal transduction pathway, the activation of AMPK will promote glucose uptake, improve IR, promote the synthesis of glycogen, and adjust energy storage and consumption. However, although early use of metformin in patients with newly diagnosed diabetes could reduce HbA1c by 1% to 2%, it fails to prevent the functional decline of pancreatic β cells. When combined with sitagliptin, the hypoglycemic effect is strengthened and also the functions of pancreatic β cells can be preserved and restored.^14,15^

Sitagliptin is cleared from the body mainly via the kidneys. Unlike patients with mild or moderate liver dysfunction, the dosage needs to be reduced in patients with kidney disorders.^16^ In our study the metformin/sitagliptin treatment did not influence kidney and liver functions differently from the metformin/glargine regimen, indicating a good tolerance.

Few reports have been published on the potential adverse reactions of sitagliptin. While 100 mg sitagliptin could suppress 97% of DPP-4 for up to 24 h, its effects on hepatic cytochrome P450 enzymes were relatively weak and its pharmacokinetic characteristics remain unaffected when used in conjunction with other hypoglycemic drugs (e.g., metformin).^17^ A therapeutic dose of sitagliptin (100 mg/d) does not increase the risk of malignancy or pancreatitis,^18^ nor will it cause gastrointestinal reactions,^4^ which is in accordance with our data of reduced gas production in the experimental group.

According to the results from our study, the effect of subcutaneous insulin injection was not as effective as that of Janumet, considering serum HbA1c reduction, quality of life improvement, and the total cost of treatment. With regard to the drug formulation, using oral FDC was simple and convenient, reduced waste, and saved resources.^19^ A retrospective cohort study showed that FDC improved patients’ compliance with regard to hypoglycemic treatment.^20^ Compared with free combination therapy, the DPP-4 inhibitors/metformin FDC provided additional hypoglycemic benefits, further reducing HbA1c by 0.25% (*P* = 0.002).^21^

The interpretations of our study are limited by the relatively short study period and the small cohort size of patients from only 2 centers. Also, diabetic complications were not included in the analysis and we did not implement the study as a double-blind trial. A future study will be needed to evaluate the chronic effects of sitagliptin/metformin therapy on T2DM patients from the pharmacoeconomical perspective, and more detailed mechanisms of the drug actions should be investigated.

## CONCLUSIONS

For overweight or obese T2DM patients whose serum glycosylated hemoglobin was >9%, oral Janumet (sitagliptin/metformin tablets) combined with low caloric diets helped to maintain glycemic control and to improve significantly the quality of life of patients, without increasing weight or adverse hypoglycemic events. This therapy was also proven to be safe, economical, and convenient.
